# Mitochondrial Genome Sequencing and Development of Genetic Markers for the Detection of DNA of Invasive Bighead and Silver Carp (*Hypophthalmichthys nobilis* and *H. molitrix*) in Environmental Water Samples from the United States

**DOI:** 10.1371/journal.pone.0117803

**Published:** 2015-02-23

**Authors:** Heather L. Farrington, Christine E. Edwards, Xin Guan, Matthew R. Carr, Kelly Baerwaldt, Richard F. Lance

**Affiliations:** 1 Environmental Laboratory, United States Army Engineer Research and Development Center, Vicksburg, Mississippi, United States of America; 2 United States Army Corps of Engineers, Rock Island, Illinois, United States of America; Natural History Museum of Denmark, DENMARK

## Abstract

Invasive Asian bighead and silver carp (*Hypophthalmichthys nobilis* and *H. molitrix*) pose a substantial threat to North American aquatic ecosystems. Recently, environmental DNA (eDNA), genetic material shed by organisms into their environment that can be detected by non-invasive sampling strategies and genetic assays, has gained recognition as a tool for tracking the invasion front of these species toward the Great Lakes. The goal of this study was to develop new species-specific conventional PCR (cPCR) and quantitative (qPCR) markers for detection of these species in North American surface waters. We first generated complete mitochondrial genome sequences from 33 bighead and 29 silver carp individuals collected throughout their introduced range. These sequences were aligned with those from other common and closely related fish species from the Illinois River watershed to identify and design new species-specific markers for the detection of bighead and silver carp DNA in environmental water samples. We then tested these genetic markers in the laboratory for species-specificity and sensitivity. Newly developed markers performed well in field trials, did not have any false positive detections, and many markers had much higher detection rates and sensitivity compared to the markers currently used in eDNA surveillance programs. We also explored the use of multiple genetic markers to determine whether it would improve detection rates, results of which showed that using multiple highly sensitive markers should maximize detection rates in environmental samples. The new markers developed in this study greatly expand the number of species-specific genetic markers available to track the invasion front of bighead and silver carp and will improve the resolution of these assays. Additionally, the use of the qPCR markers developed in this study may reduce sample processing time and cost of eDNA monitoring for these species.

## Introduction

Invasive aquatic nuisance species pose a major threat to aquatic ecosystems worldwide. In North America, invasive Asian carps, particularly bighead carp (BHC; *Hypophthalmichthys nobilis*) and silver carp (SC; *H*. *molitrix*), have been very problematic in freshwater ecosystems. Asian carps were imported into the U.S. in the 1970s to control algae in Arkansas fish farms [1]. Flooding allowed them to escape and establish reproducing populations in the wild by the early 1980s. They have since been steadily dispersing upstream throughout the Mississippi River watershed [1,2]. At present, BHC and SC have been found in 23 states, and they have rapidly expanded their population sizes, with BHC and SC representing over 60% of the biomass in some portions of their North American range [3]. These filter feeders cause significant ecological impacts by altering plankton communities at the base of the food chain and outcompeting native species for resources [4,5]. There is considerable concern that these species will enter the Great Lakes through man-made shipping, sanitation and flood control canals, such as those of the Chicago Area Waterways System (CAWS). Although it is impossible to fully predict the potential effects of self-sustaining populations of BHC or SC in the Great Lakes, there is concern that these species have the potential to dramatically alter the ecosystem, leading to negative effects on populations of native fishes and many threatened or endangered plant/animal species [6]. The impact of this invasion on Great Lakes commercial and recreational fisheries is of particular concern.

Aquatic organisms shed biological materials (*e*.*g*., scales, epithelial cells, slime coats, waste) containing DNA into their environments. This environmental DNA (eDNA) can persist in aquatic environments for extended periods (range 7–30 days) [7–11], and the eDNA in water samples can be assayed using species-specific genetic markers to determine whether a species of interest may be present. Because eDNA can be detected in water when target species’ populations are at low abundances, eDNA techniques may be particularly helpful in tracking changes in the distributions of aquatic invasive species [7,12–18] or identifying locations where threatened or endangered species may occur [19–21].

Since 2009, eDNA monitoring has been used to track the invasion front of BHC and SC throughout the CAWS, Des Plaines River, and near-shore waters of Lake Michigan [6]. The use of eDNA monitoring is specified for use by the Asian Carp Regional Coordinating Committee in the Asian Carp Monitoring and Rapid Response Plan, and represents a collaborative effort by the United States Fish and Wildlife Service (USFWS), the United States Environmental Protection Agency (USEPA), the United States Army Corps of Engineers (USACE), and various state agencies. Independent monitoring efforts for BHC and SC have also been carried out by the University of Notre Dame [13–14]. The current eDNA monitoring program utilizes conventional polymerase chain reaction (cPCR) analysis, whereby the presence or absence of a particular species’ DNA is determined by PCR amplification of a target DNA fragment of the mitochondrial genome [13]. The PCR-amplified product is then isolated by gel electrophoresis and the DNA is sequenced to confirm the species of origin. The Quality Assurance Project Plan (QAPP) for the Environmental DNA (eDNA) Monitoring of Invasive Asian Carp in the CAWS outlines the detailed procedures for the current planning, collection, filtering and processing of eDNA samples [22].

The development of additional BHC and SC eDNA markers could provide a suite of assays to provide multiple lines of evidence or secondary verification for eDNA detections. In addition to cPCR markers, quantitative PCR (qPCR) may be used as an eDNA monitoring tool. The use of qPCR has several potential advantages relative to cPCR, including, typically, more rapid PCR thermal-cycling programs, which can be important for large-scale sampling efforts, a reduced sensitivity in some cases to PCR inhibitors [personal observation; 10], and the ability to quantify the amount of DNA in a sample [23]. Also, while conventional PCR requires specific oligonucleotide binding at *two* locations (the forward and reverse primers) to produce a PCR product, hydrolysis probe-based qPCR, which is one of two common qPCR methodologies, may often be a more stringent assay because it requires specific oligonucleotide binding at *three* locations (forward and reverse primers, as well as the internal hydrolysis probe) in order for the reaction to produce a product that emits a fluorescent signal [24–25].

Our objectives in this study were to: 1) sequence full mitochondrial (mtDNA) genomes from multiple BHC and SC throughout their North American range to represent the intraspecific genetic variation of each species, 2) use multiple sequence alignments of BHC, SC and other closely related non-target species that may be present in aquatic ecosystems in the Midwestern U.S.A. to design species-specific cPCR and qPCR markers for the detection of BHC and SC in eDNA monitoring programs, and 3) test the specificity and sensitivity of these new markers in detecting BHC and SC in laboratory and eDNA field trials. We also explored whether using combinations of multiple markers would improve the sensitivity and detection rates in eDNA monitoring programs for SC and BHC.

## Methods

### Ethics Statement

Prior to 2014, the IACUC at the US Army ERDC was focused exclusively on on-site lab-based research, so we were unable to obtain IUCAC approval for this study because we lacked an appropriate IUCAC authority to approve our field-based collections. Therefore, all whole fish and fish tissue sample collection followed guidelines provided by the American Society for Ichthyologists and Herpetologists’ (ASIH) Guidelines for the Use of Fishes in Research (American Fisheries Society 2004), including, in order to minimize stress and suffering, rapid euthanasia via a forceful blow to the top rear margin of the cranium or pithing soon after removal from the water. All invasive species were handled in accordance with the provisions mandated by the Lacey Act and the Asian Carp Prevention and Control Act. Silver and bighead carp were collected and euthanized from the middle and lower Mississippi under authorities granted by state scientific collecting permits for Arkansas (#053120121), Louisiana (#118), and Mississippi (#0827121). Silver carp, bighead carp, and all fish used in cross-species amplification trials were collected from the Illinois River and Chicago Area Waterway System by the Illinois Department of Natural Resources under statutory authority granted under Illinois Fish and Wildlife Statutes 515 ILCS 5/20–100 and 520 ILCS 5/3.22.

### Sample Collection, DNA sequencing, and Alignment

**Fig 1:**
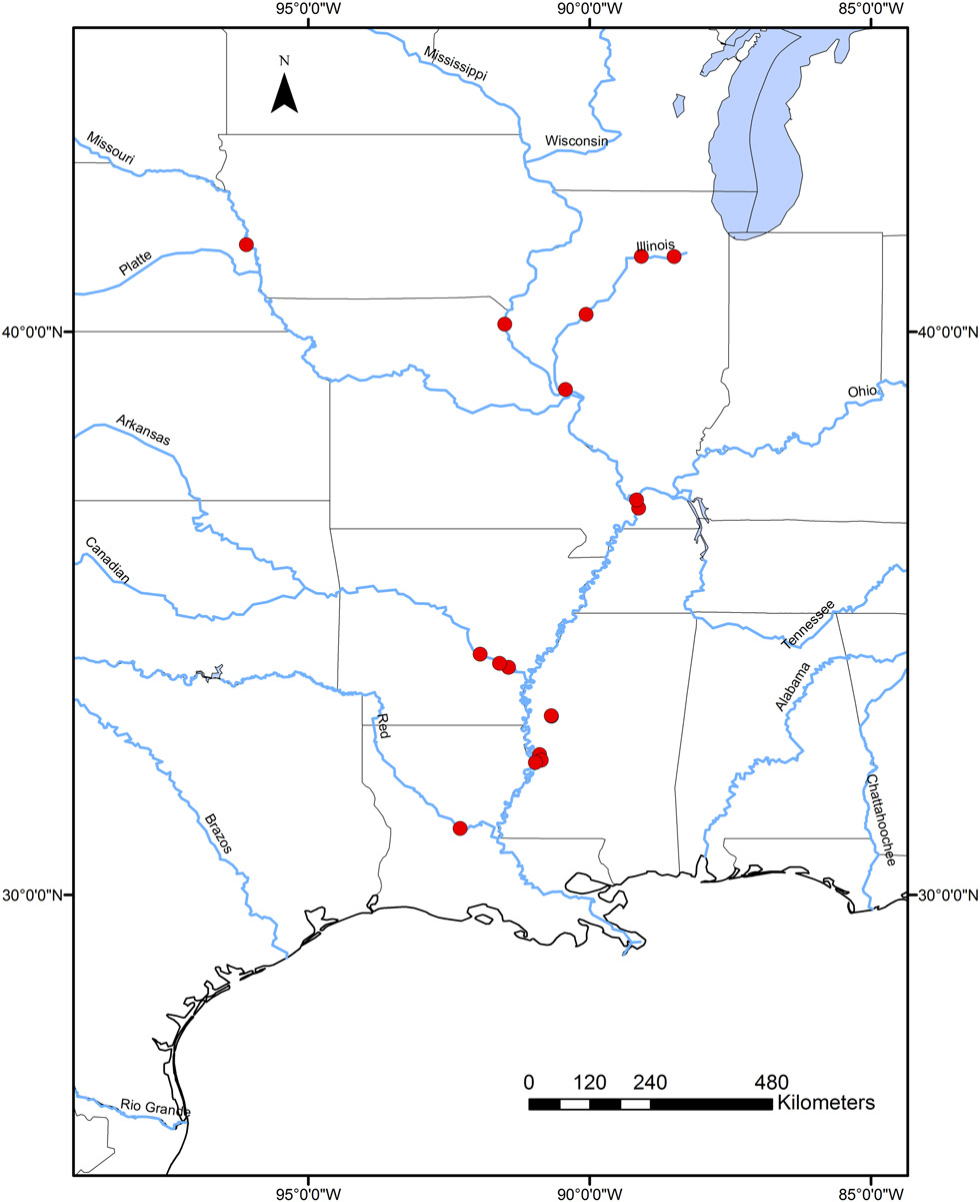
Map of Sampling Locations. Geographic distribution of sample collection for mitochondrial DNA sequencing.

Tissue samples (fin clips or livers) were collected from SC and BHC populations throughout their introduced range within the Mississippi River watershed (Table 1; Fig. 1). Individuals were identified based on morphology in the field, and we collected samples only from individuals that could be positively identified as either BHC or SC. Although BHC and SC hybridize readily in the North America [26], we did not collect samples from putative hybrid individuals of intermediate morphology. Samples were placed in individual containers in 95% ethanol or RNAlater (Qiagen) for subsequent DNA extraction in the lab.

**Table 1:** Sample Information.

Location	Silver Carp (n)	Silver Carp GenBank accession #	Bighead Carp (n)	Bighead Carp GenBank accession #
East Lower Mississippi (Yazoo River, Steele Bayou, Big Sunflower River)	3	KJ746964, KJ746965, KJ746946	5	KJ729086, KJ729095—KJ729097, KJ746959
West Lower Mississippi (Red River, Atchafalaya River)	3	KJ746947—KJ746949	6	KJ729084, KJ729085, KJ729087—KJ729089, KJ746963
Arkansas River	3	KJ671449, KJ671450, KJ746956	3	KJ729081—KJ729083
Ohio River (at junction to Mississippi River)	3	KJ746938—KJ746940	3	KJ746935—KJ746937
Mississippi River (Knowlton Lake)	2	KJ679503, KJ746961	-	
Illinois River (LaGrange Reach)	-		3	KJ679504, KJ679505, KJ710362
Illinois River (Marseilles Reach)	3	KJ729076, KJ746953, KJ746960	3	KJ710363, KJ729079, KJ729080
Illinois River (Starved Rock)	2	KJ746954, KJ746957	3	KJ729090, KJ746966, KJ756343
Mississippi River (Laketon, KY)	3	KJ729092—KJ729094	1	KJ729091
Upper Mississippi River (Pool 20)	1	KJ746955	3	KJ729077, KJ729078, KJ746958
Upper Mississippi River (Pool 26)	3	KJ746943—KJ746945	3	KJ746941, KJ746942, KJ746962
Missouri River (north of Omaha)	3	KJ746950—KJ746952	-	

Origins of silver and bighead carp samples included in mtDNA genome sequencing and GenBank accession numbers for the sequences generated from the individuals at each sampling location.

Total genomic DNA was extracted using DNeasy Blood and Tissue Kits (QIAGEN Inc.) according to the manufacturer’s instructions. DNA extractions were enriched for mitochondrial DNA using long PCR to amplify the mitochondrial genome as a single 16.6 kb fragment. Primer sequences were S-LA-16S-L 5´-CGATTAAAGTCCTACGTGATCTGAGTTCAG-3´ and S-LA-16S-H 5´-TGCACCATTAGGATGTCCTGATCCAACATC-3´ [27]. QIAGEN LongRange PCR Kit reagents were used to formulate a 25 μL PCR reaction mixture containing 1× LongRange PCR buffer, 500 μM dNTPs, 1.25 U LongRange PCR Enzyme mix, 0.4 μM of each primer, and 1 μL of DNA template. Temperature cycling conditions began with an initial denaturation step of 93°C for 3 min, followed by 10 cycles of 93°C for 15 sec, 62°C for 30 sec and 68°C for 18 min. An additional 29 cycles were then run, adding 20 sec to the extension step for each cycle. Because amplification of a single fragment was not successful for all samples (likely due to degraded template DNA), we also attempted to amplify the mitochondrial genome in three shorter, overlapping fragments, using the same PCR chemistry and cycling conditions described above. Primer sequences were designed using Primer3 software [28] based on BHC and SC complete mitochondrial genome sequences available on GenBank (accession numbers NC_010194, EU343733, JQ231114, HM162839, EU315941, NC_010156). The following primers were designed to amplify fragments of approximately 7.4, 7.0 and 3.0 kb, respectively: LC1-F and R (5´ - GAATGGGCTAAACCCCTAAA -3´ / 5´- TCGTAGTGAAAAGGGCAGTC -3´); LC2-F and R (5´ - CAGGATTCCACGGACTACAC -3´ / 5´ - TTGGGGTTTGACAAGGATAA -3´; LC3-F and R (5´ - CATGCCGAGCATTCTTTTAT -3´ / 5´ - CAACATCGAGGTCGTAAACC- 3´). When agarose gel electrophoresis revealed that all three of the shorter PCR reactions produced bands of the expected sizes, the reaction products were pooled for sequencing.

PCR products were purified using ZR-96 DNA Clean and Concentrator-5 kits (Zymo Research) and prepared for next-generation sequencing using Nextera DNA Sample Preparation Kits (Illumina, Inc.). A separate library was prepared for each sample (totaling 29 libraries for SC and 33 libraries for BH), and Nextera Index Kits were used to pool libraries into runs for sequencing. Sequencing was performed on the Illumina MiSeq system, using 150 bp paired-end reads. MiSeq Reporter Software was used to sort the resulting pool of sequences by the indices to identify the sequences arising from each sample. Mitochondrial genomes were assembled by aligning the reads of each individual to a reference sequence of the appropriate species from GenBank (EU315941.1 for SC and EU343733.1 for BHC) using the medium sensitivity/fast settings in Geneious software v.6 (Biomatters Ltd., Auckland, New Zealand). Consensus sequences generated for each individual were exported and aligned, along with sequences of some related cyprinid fish species that may be present in the same North American regions as BHC and SC (common carp, grass carp and black carp; GenBank accession numbers NC_010288.1, NC_018035.1, NC_018039.1, NC_018036.1, NC_011141.1). Alignments were carried out using the default settings in MUSCLE [29] as implemented in Geneious V6.

### Marker Design

Marker loci were designed using the multiple sequence alignment of complete mitochondrial genomes of BHC, SC, and several related species (listed above). Potential PCR primer sites were chosen by identifying sequence regions that demonstrated no mismatches within the target taxa and that maximized differences between target and non-target taxa. Because eDNA may experience rapid degradation by environmental conditions, marker loci were designed to be short (<400 bp) to increase amplification probability. Primer3 [28] was used to design cPCR primers and qPCR primer/probe sets with preference for primers that contained 3′-end mismatches to homologous DNA in non-target species. All qPCR probes were labeled with 6FAM as the 5′ fluorescent tag, and TAMRA as the 3′ quencher. Due to the limited genetic divergence between bighead carp and silver carp, we also developed a series of general markers, designated as “AC”, for detection of both species.

### Marker Testing for Specificity and Efficacy in eDNA Field Trials

Unless otherwise noted, all assays to test newly designed cPCR markers used 25 μL reactions containing 1× Platinum *Taq* PCR buffer (Invitrogen), 200 μM dNTPs, 1.5 mM MgCl_2_, 0.2 μM of each primer, 1.25 U Platinum *Taq* polymerase (Invitrogen), and 1 μL DNA template. Temperature cycling conditions began with an initial denaturation step of 94°C for 10 min, followed by 45 cycles of 94°C for 1 min, 52°C for 1 min and 72°C for 1 min 30 sec, with a final elongation step at 72°C for 7 min. Forty-five cycles of PCR were conducted for consistency with current eDNA monitoring protocols to allow for direct comparison of current and newly-developed cPCR markers. Amplification products of cPCR assays were purified using E-Gel SizeSelect Gels (Life Technologies) and sequenced directly using an ABI 3500XL Genetic Analyzer with BigDye chemistry and standard sequencing protocols. To identify the source species of the amplification product, resulting sequences were compared against BHC and SC reference DNA sequences and subjected to GenBank BLAST searches.

All qPCR reactions were run in 20μL volumes containing 1X TaqMan Environmental Master Mix, 0.5 μM of each primer, 0.125 μM of the probe, and 1μL of DNA template. Temperature cycling began with an initial denaturation step at 95°C for 10min, followed by 40 cycles of 95°C for 15 sec and 60°C for 1 min. qPCR reactions were run on a ViiA 7 Real-Time PCR System (Applied Biosystems). qPCR reactions were considered positive if the amplification curve crossed the fluorescence detection threshold by the end of the 40 cycle qPCR run. Because there are no current qPCR markers or methodology for Asian carp monitoring for direct comparison to new markers, we used the industry standard thermocycling protocol of 40 cycles typically used in TaqMan assays for qPCR marker development.

Both cPCR and qPCR markers were tested for: 1) species-specificity, 2) ability to amplify target species DNA from eDNA samples collected in areas of known BHC and SC presence, 3) false-positive amplification from eDNA samples that likely do not contain Asian carp DNA, and 4) limits of detection, or sensitivity, in targets species (*i*.*e*., the minimum amount of starting DNA that can result in a detectable cPCR or qPCR product).

Species-specificity of both cPCR and qPCR assays was tested using a panel of genomic DNA (normalized to 1 ng/μL) from individuals of the target species and 29 additional species likely to be present in the CAWS, using the methods for cPCR or qPCR described at the beginning of the section. This panel included closely related, non-target species such as shiners, common carp, goldfish, and grass carp (Table 2). If cPCR markers amplified non-target species, annealing temperatures were adjusted in an attempt to eliminate non-target amplification.

**Table 2:** Non-target Fish Species Test Panel.

Common Name	Species name
Brown Bullhead	*Ameiurus nebulosus*
Freshwater Drum	*Aplodinotus grunniens*
Goldfish	*Carassius auratus*
Quillback	*Carpiodes cyprinus*
Grass Carp	*Ctenopharyngodon idella*
Spotfin Shiner	*Cyprinella spiloptera*
Common Carp	*Cyprinus carpio*
Gizzard Shad	*Dorosoma cepedianum*
Channel Catfish	*Ictalurus punctatus*
Smallmouth Buffalo	*Ictiobus bubalus*
Black Buffalo	*Ictiobus niger*
Brook Silverside	*Labidesthes sicculus*
Green Sunfish	*Lepomis cyanellus*
Pumpkinseed Sunfish	*Lepomis gibbosus*
Orangespotted Sunfish	*Lepomis humilis*
Bluegill	*Lepomis macrochirus*
Smallmouth Bass	*Micropterus dolmieu*
Largemouth Bass	*Micropterus salmoides*
White Perch	*Morone americana*
White Bass	*Morone chrysops*
Round Goby	*Neogobius melanostomus*
Golden Shiner	*Notemigonus crysoleucas*
Emerald Shiner	*Notropis atherinoides*
Yellow Perch	*Perca flavescens*
Bluntnose minnow	*Pimephales notatus*
White Crappie	*Pomoxis annularis*
Black Crappie	*Pomoxis nigromaculatus*
Flathead Catfish	*Pylodictis olivaris*

Panel of 29 non-target fish species collected from the CAWS used for testing of primers for cross-species amplification. No amplification of these species was observed in the markers presented in Table 3.

**Table 3:** Primer Information.

Name	Target species	Type of assay	Forward	Reverse	Probe (TaqMan markers only)	Length (bp)
SC-1	SC	cPCR	GGACCCAGTACTATTAACTGCTCTA	TCCTAGGGCAAGGAGGGTA		171
SC-5	SC	cPCR	TCCGATTACCGCCACAATTATAGCCTTAG	GATAGGGTTAGTGGAAGAGAGGAC		161
SC-7	SC	cPCR	ACTGAATAAACACACACATGTTCGAT	ATCATCACCCGATTAGTAAAAATG		275
SC-TM4	SC	qPCR	CCACTAACATCACCACGCAA	AGCCTTTTCCAGAGGCTTGG	TAACCCAGCTGCCAATACAA	168
SC-TM5	SC	qPCR	CCACAACTTACCCTCCTTGCC	AAGGGTATTAATTTTTGTGGTGGA	TCATGACATCCGCAGCATTCCTC	98
BH-6	BHC	cPCR	CAATACCCTAGCAATTATTCCCTTA	TGTAATTCCAAGGGCGGTTAG		375
BH-8	BHC	cPCR	gatgtaaactatggctggcttatt	tgtagaaagaggaggtgtaggaa		388
BH-TM1	BHC	qPCR	TAGACCTTCTAACAGGACTAATTC	AATCCACCTCATCCTCCAAC	CCGCCCTTGGAATTACATCCACA	144
BH-TM2	BHC	qPCR	CCTTCGTCAAACAGACCTTAAATCC	CCCTCATGGGGTTTGGATTAGA	CCACATAGGACTTGTAGCGGGTGGA	96
BH-TM4	BHC	qPCR	CCACTAACATCGCCACGTAG	AACCTTTTCCAGAAGCTTGG	TAGCCCAGCCGCCAACACAA	168
AC-6	Both	cPCR	GTTCCTAATCAGCACCTTAGTACTCT	AATTCGAAGGGATGGCAAG		156
AC-TM1	Both	qPCR	GGCCGGAACAGGATGAACAGTT	TAATAGTTGTGGTGATGAAGTTAATTG	CACGCAGGAGCATCCGTAGACCT	145
AC-TM2	Both	qPCR	CAATTAACTTCATCACCACAACTATTA	TCCAGCAGCTAAAACTGGTAAGG	AAACACCTCTCTTTGTTTGAGCTGTGC	133
AC-TM3	Both	qPCR	TTCATCGGCGTAAATCTTACAT	AGGGAAATAAGAGATCCGATAGA	ACCCAGATGCCTACGCCCTG	133

Primer sequences of markers used for field and sensitivity testing. For targeted species, BHC = bighead carp, SC = silver carp, Both = primers that could potentially be used for non-specific detection of both BHC and SC.

The cPCR and qPCR markers that amplified the target species and showed no cross-amplification in non-target species were further tested using field-collected water samples to evaluate the detection rate of BHC and SC from areas of known presence and the potential false positive rate from waters where they are absent. To obtain samples from areas of known presence of both BHC and SC, we collected samples from Steele Bayou, a backwater flood-control area near the Yazoo River’s confluence with the Mississippi River near Vicksburg, MS., U.S.A. Steele Bayou is locally known to have well established BHC and SC populations with high densities (pers. comm, A. Katzenmeyer). To obtain samples where BHC and SC are absent, we collected water samples from a small tributary of Fishing Creek (Clinton County, PA, U.S.A), an area outside the introduced range of these species. These samples have all the typical components of environmental water samples, but were free of DNA of the target species, and thus provided test cases to detect potential non-target amplification within naturally occurring DNA pools. Water samples were collected from the water surface film in 50 mL conical tubes during June-July 2013. In the laboratory, tubes were centrifuged at maximum speed (4000 g) for 30 min at 4°C. The supernatant was poured off and DNA was extracted from the remaining pellet of material using a modified cetyltrimethyl ammonium bromide (CTAB)/chloroform protocol [30]. Each cPCR and qPCR marker was tested, using the methods listed at the beginning of the section, on a panel of 44 Steele Bayou and 44 Fishing Creek samples, with 4x replication of PCR reactions. For these samples and assays, we employed strict quality control measures to avoid sample contamination as outlined in [22], including physical separation and different instruments for pre- and post-PCR sample processing, use of a PCR hood with UV and HEPA filter, and negative controls. We scored a marker as positive for a sample if we obtained a positive result for the target species (sequence-confirmed in the case of cPCR, or detection of fluorescent signal above the determined threshold in the case of qPCR) for at least one of the four PCR replicates. The performance of all new markers, as measured by rate of detection across the positive and negative samples, was compared to the cPCR markers for BHC and SC from [14] (primers HN203-F: TAACTTAAATAAACAGATTA & HN498-R: TAAAAGAATGCTCGGCATGT, and HMF-2: CCTGARAAAAGARKTRTTCCACTATAA & HMR-2: GCCAAATGCAAGTAATAGTTCATTC, respectively), which are currently used in the Asian carp monitoring QAPP [22]; we refer to these markers here as QAPP-SC and QAPP-BH.

Markers with high detection rates and no false-positive detections in environmental samples were subjected to sensitivity testing. Genomic DNA of SC and BHC was extracted and the concentration of each was normalized to 1 ng/μL. A serial 1:10 dilution series was prepared and markers were tested, using the methods described at the beginning of the section, across the concentration range of 0.1 ng/μL (10^-1^) through 10^-7^, with four replicate cPCRs or qPCRs at each concentration. A limitation to the use of genomic DNA in sensitivity testing for cPCR markers is that the number of marker copies present in the normalized DNA extractions, and therefore available for PCR amplification, is unknown. To estimate starting copy number in qPCR reactions, each qPCR marker was cloned into a bacterial plasmid vector using TOPO Cloning kits (Life Technologies) as per the manufacturer’s instructions. Successfully cloned bacterial colonies were cultured and plasmids extracted using Qiagen Miniprep plasmid extraction kits. The estimated number of plasmids in the resulting elutions was calculated using the combined base pair length of the plasmid and marker insert, a standard DNA base-to-Daltons conversion for double-stranded DNA (650 Daltons/base) [31], a Daltons-to-nanograms conversion, and DNA mass quantification of elutions using a NanoDrop 1000. A dilution series of the plasmid elution was then used to generate a standard curve for estimation of copy number in the qPCR reactions (S1 Table). Circular plasmid standards are expected to be effective PCR standards for loci found on circular DNAs, like mitochondrial genomes [32].

To test whether the throughput of eDNA screening methods could be increased by assaying for multiple markers simultaneously within single PCRs, several markers were combined in pairs for multiplex qPCR reactions. Three primer sets were tested: BH-TM1/BH-TM2, SC-TM4/SC-TM5, and AC-TM1/AC-TM3. For qPCR multiplexing, the two markers utilized probes with different fluorescent labels (FAM or VIC). A genomic DNA dilution series and plasmid standards were again used for testing, with markers and standards run both individually and in combination in order to directly compare sensitivity in single versus multiplex reactions. qPCR reactions were prepared as described at the beginning of the section, with both sets of primers and probes added to the reaction, and the same temperature cycling conditions. We did not multiplex cPCR markers because they were all designed to be in the size range of 200–300 base pairs to increase the potential for amplification of degraded eDNA, such that amplicon base pair lengths were too similar for clear differentiation of bands on 2% agarose gels.

## Results

We generated complete mtDNA sequences for 33 BHC and 29 SC individuals; all DNA sequences were submitted to GenBank (See Table 1 for collection location information and GenBank Accession numbers). Average whole genome sequence coverage was 1595X (range 3–11971X coverage). Total length of the aligned BHC and SC genomes was 16,620 bp. There was very little sequence variation within species, with only 40 (0.24%) and 34 (0.20%) variable sites for BHC and SC, respectively. When species alignments were combined, there were a total of 823 (4.95%) variable sites across the mitochondrial genome. These genomes were aligned with mtDNA genomes from closely related species obtained from GenBank, including common, grass and black carp for identification of potential species-specific eDNA markers.

Based on the alignment of mitochondrial genomes, we initially designed 12 SC, 11 BHC and 16 general (AC) cPCR markers. For TaqMan qPCR, we initially designed five markers for BHC, six for SC, and three general (AC) markers. Markers were located throughout the mitochondrial genome, wherever we found suitable patterns of variation to design species-specific markers. Based on results from the initial cross-species screening, several markers amplified non-target species and were not further investigated, reducing the number of potential markers for testing to six for cPCR and eight for qPCR (Table 3). We focused all subsequent field and sensitivity testing on the markers with high affinity for the target species and no amplification of other species.

Assays of the 44 Steele Bayou samples with the established markers QAPP-SC and QAPP-BH resulted in 28 (64%) positive SC detections and 0 (0%) positive BHC detections. All of the newly designed BHC markers performed better than the QAPP-BH marker (Table 4), with the number of positive BHC detections ranging from 6 to 9; the highest detection rate was from the qPCR marker BH-TM2 (9 of 44, 20%). In comparison to the QAPP-SC marker, all newly-designed SC markers had similar or higher numbers of detections, with the number of positive SC detections ranging from 23 to 32; the highest detection rates were from cPCR marker SC-1 and qPCR marker SC-TM5, both with 32 positive samples (73%). Positive detection rates were 57–68% for the general (AC) markers. For the Fishing Creek samples, none of the new cPCR markers produced bands in the same size range as target species and none of the qPCR markers produced quantifiable fluorescence.

**Table 4:** Summary Data from Field Testing.

Marker name	Type of assay	Steele Bayou	Fishing Creek
**Silver Carp:**			
QAPP-SC	cPCR	28	0
SC-1	cPCR	32	0
SC-5	cPCR	25	0
SC-7	cPCR	23	0
SC-TM4	qPCR	26	0
SC-TM5	qPCR	32	0
**Bighead Carp:**			
QAPP-BH	cPCR	0	Not tested
BH-6	cPCR	6	0
BH-8	cPCR	6	0
BH-TM1	qPCR	9	0
BH-TM2	qPCR	7	0
BH-TM4	qPCR	6	0
**Bighead and Silver Carp:**			
AC-6	cPCR	30	0
AC-TM1	qPCR	25	0
AC-TM2	qPCR	30	0
AC-TM3	qPCR	28*	0

*—Tested with only 42 samples

Number of positive detections noted for each marker tested using 44 eDNA field samples. Steele Bayou samples were collected from an area of high concentrations of both BHC and SC, whereas Fishing Creek samples were collected from an area where carp are absent. QAPP-SC and QAPP-BH are the markers currently used for eDNA testing. Names containing TM indicate TaqMan qPCR markers.

To understand how using a combination of markers may improve detections, we investigated the patterns of amplification across markers for each of the 44 Steele Bayou samples. For SC, the highest number of detections for a single marker was only 32 for both SC-1 and SC-TM5 (S2 Table), while a combination of these two SC markers resulted in positive detections in all 38 of the samples that were positive for at least one marker (S2 Table). For BHC, the highest number of detections for a single marker was only 9 (BH-TM1; S3 Table), while using a combination of the two best BHC markers (BH-TM1 and BH-TM2) resulted in detection of DNA for this species in 15 of the 23 samples that were positive for at least one marker (S3 Table). For the AC markers, the highest detection rate for a single marker was 30 samples in both AC-6 and AC-TM2, while using a combination of these two AC markers resulted in positive detections in 37 of the 40 samples that were positive for at least one marker (S4 Table). Overall, these results show that using multiple markers will dramatically improve detection rates for BHC and SC eDNA assays.

All the tested markers consistently yielded positive results from genomic DNA down to at least the 10^-3^ dilution (0.001 ng/μL). Three SC (SC-5, SC-7 and SC-TM4) and one BHC/SC marker (AC-TM2) had consistent detections at 10^-4^, and nearly all markers had >50% detection rates among the four replicates at the 10^-4^ dilution level, which is estimated to have copy numbers in the single digits (Table 5). Detections became more stochastic at concentrations below 10^-4^, as expected for samples with extremely low copy number (average of ≤1 marker copy per reaction). Sensitivity of the new BHC and SC markers was comparable to the QAPP markers.

**Table 5:** Sensitivity Trials.

Marker	Type of assay	Dilution:						
		10^-1^	10^-2^	10^-3^	10^-4^	10^-5^	*10^-6^	*10^-7^
**Silver Carp:**								
QAPP-SC	cPCR	1.00	1.00	1.00	1.00	0	0	0
SC-1	cPCR	1.00	1.00	1.00	0.75	0	0	0
SC-5	cPCR	1.00	1.00	1.00	1.00	0	0.50	0
SC-7	cPCR	1.00	1.00	1.00	1.00	0	0	0
SC-TM4	qPCR	1.00	1.00	1.00	1.00	0.25	0.25	0
SC-TM5	qPCR	1.00	1.00	1.00	0.75	0.50	0.25	0.25
*Estimated Target-Sequence Copy Number*:		6051	508	46	3.4	1.6	U	U
**Bighead Carp:**								
QAPP-BH	cPCR	1.00	1.00	1.00	1.00	0.25	0.25	0
BH-6	cPCR	1.00	1.00	1.00	0.25	0	0	0
BH-8	cPCR	1.00	1.00	1.00	0.75	0	0.25	0
BH-TM1	qPCR	1.00	1.00	1.00	0.75	0.25	0	0
BH-TM2	qPCR	1.00	1.00	1.00	0.75	0	0	0
BH-TM4	qPCR	1.00	1.00	1.00	0.75	0	0.25	0
*Estimated Target-Sequence Copy Number*		2193	230	16	2.2	1.1	U	U
**Bighead and Silver Carp:**								
AC-6	cPCR	1.00	1.00	1.00	0.50	0	0.25	0
AC-TM1	qPCR	1.00	1.00	1.00	0.50	0.50	0	0
AC-TM2	qPCR	1.00	1.00	1.00	1.00	0.50	0	0
AC-TM3	qPCR	1.00	1.00	1.00	0.75	0	0	0
*Estimated Target-Sequence Copy Number*		6051	508	46	3.4	1.6	U	U

Sensitivity testing of each cPCR and qPCR marker across a dilution series of genomic DNA. The proportion of detections out of four replicates is noted for each marker at each dilution level. Estimated marker copy numbers per dilution are noted below each set of markers based on averages calculated across all replicates of qPCR sensitivity trials using a plasmid DNA standard. AC markers were tested using SC dilutions. U = Undeterminable copy number.

*Variation in amplifications at these levels are likely due to stochasticity of PCR at such low DNA concentrations.

Trials of multiplexed qPCR markers were successful for all combinations of markers tested, with no substantial reduction in marker sensitivity (limits of detection; Table 6).

**Table 6:** Multiplex qPCR Data.

Target Species	Trial	Marker	Dilution:			
			10^-2^	10^-3^	10^-4^	10^-5^
**Silver Carp**	Single	SC-TM4	30.6 (195–424)	34.1 (10–50)	37.3 (0–12)	38.7 (0–2.4)
	Single	SC-TM5	30.4 (872–1498)	33.7 (60–208)	37.4 (0–30)	38.1 (0–11)
	Multiplex	SC-TM4	30.7 (203–422)	34.2 (11–50)	37.9 (0–5)	38.4 (0–3)
	Multiplex	SC-TM5	30.4 (1145–1792)	33.9 (81–200)	37.5 (2–23)	38.4 (0–14)
**Bighead Carp:**	Single	BH-TM1	31.2 (107–244)	34.6 (8–28)	38.2 (0–4)	39.0 (0–1)
	Single	BH-TM2	31.2 (120–216)	34.8 (7–19)	37.8 (0–4)	39.3 (0–1)
	Multiplex	BH-TM1	30.6 (118–263)	34.3 (8–23)	37.3 (0–5)	38.3 (0–1)
	Multiplex	BH-TM2	31.1 (137–201)	34.6 (12–24)	38.1 (0–7)	38.7 (0–1)
**Bighead and Silver Carp:**	Single	AC-TM1	31.1 (241–358)	34.5 (16–65)	38.0 (0–12)	38.9 (0–3)
	Single	AC-TM3	29.1 (225–398)	32.3 (16–52)	36.0 (0–6)	37.7 (0–1)
	Multiplex	AC-TM1	31.1 (283–536)	34.5 (22–91)	37.7 (0–16)	39.4 (0–1)
	Multiplex	AC-TM3	29.1 (252–501)	32.4 (19–80)	35.9 (0–12)	37.6 (0–2)

Results of multiplexing trials of qPCR markers. Average Ct values, with the range of copy numbers in parentheses, found across 24 replicates for markers run singly or combined in a multiplex reaction.

## Discussion

The large number of mtDNA haplotypes generated in this study allowed us to capture inter- and intra-species genetic variation in SC and BHC across their introduced range in North America. This information, along with comparisons to DNA sequences from related species found in the central United States, aided in the design of cPCR and qPCR markers specifically for eDNA testing for SC and BHC in their introduced range. Effective design of PCR-based assays for the differential or discriminatory detection of species requires that sequence differences among taxa be clustered so that multiple differences among taxa are grouped into the length of a PCR primer and two or more of these areas are grouped within a few hundred base pairs. Because SC and BHC are closely related and have very low levels of sequence divergence across their mitochondrial genomes, a very limited number of sites demonstrated a sufficient number of clustered polymorphism to develop effective species-specific markers. Despite careful selection of markers to maximize differences among species, cross-amplification was observed in at least one non-target species for many markers, resulting in the elimination of nearly 70% of the originally designed markers. Despite these difficulties, we were able to design multiple cPCR and qPCR markers that specifically detect SC and BHC in field-collected water samples in North America.

In field trials, the new species-specific BHC markers yielded more positive detections than the QAPP-BH marker. Although the QAPP-BH marker performs well in genomic DNA sensitivity tests, this marker failed to detect DNA of BHC in any environmental sample from an area of known BHC occurrence (Table 1). These results are similar to those of the eDNA monitoring in the CAWS and greater Midwestern region from 2010–2014 [33–34], in which the QAPP-BH marker detected DNA of BHC in only 2 environmental samples. One potential reason why the QAPP-BH may perform so poorly is that the basic melting temperature for this marker is 37C, which is well below the annealing temperature of 50C used for this assay [14,22]. In contrast, all the newly designed markers for detection of bighead carp generated positive detections of BHC DNA in environmental samples from Steel Bayou, and clearly represent a major improvement over the QAPP-BH marker. The SC markers developed in this study generally had detection rates similar to or higher than the marker currently used (QAPP-SC) to detect the presence of SC DNA in environmental water samples, with similar levels of sensitivity at low concentrations of target DNA.

For both species, confounding or efficiency-diminishing factors (e.g., non-specific amplicons that result in gel bands of similar size to those obtained for the target species or nontarget fluorescence in qPCR trials) were not observed, indicating that these markers would be ideal for high-throughput assays to detect the presence of BHC and SC from environmental water samples. Multiplexing of qPCR markers was successful in genomic DNA trials, suggesting that multiplexing may be feasible in eDNA screening, increasing throughput of the assays. However, performance of multiplexing reactions with field eDNA samples remains to be tested, and additional combinations of the various markers could be employed following further testing. The addition of qPCR technology to eDNA screening provides the transition from simple presence/absence data provided by cPCR to the generation of data related to DNA concentration in field samples. This additional information may help estimate relative abundance or biomass of species of interest in the sampled waterway. qPCR may also reduce sample screening time by eliminating the need for gel electrophoresis and sequence verification.

In addition to potential improvements in sensitivity and throughput by the markers developed in this study, the availability of multiple new PCR markers for eDNA screening of SC and BHC in water samples may help increase the accuracy of eDNA monitoring programs. eDNA samples are largely comprised of randomly fragmented, low-abundance DNA targets. The current program uses a single marker locus to detect the presence of SC or BHC in environmental samples, which may be sensitive to random degradation of the single marker. In this study, using a combination of two markers in eDNA field trials showed improved overall detection rates and provided stronger evidence for the presence of BHC and SC DNA in the water. One possible reason for the increase in detections using multiple markers is simply that a greater number of PCR reactions were carried out, increasing the likelihood that a fragment of BHC or SC DNA was present in at least one PCR reaction. However, another possibility is that DNA of SC or BHC may be present in the water, but because degradation may occur at one marker locus but not others, using multiple loci would help to overcome false negatives due to stochastic degradation in a single marker locus. In reality, it is likely that both increased numbers of PCR reactions and the use of multiple markers to ensure against the stochastic process of DNA degradation are likely to have increased detection rates. Regardless, because use of a combination of markers appears to be a much more highly sensitive way to detect eDNA in environmental samples, we therefore recommend that eDNA detection programs employ the use of a combination of two or more markers in the future.

Although the markers developed in this study performed well in trials to detect either BHC or SC, one concern is how eDNA data would be interpreted in the presence of interspecific hybridization, as hybridization between BHC and SC is common in many North American populations [26]. The species-specific eDNA markers developed in this study could, in some cases, detect interspecific hybrids, although they would not be recognized as a hybrid because mtDNA is inherited maternally. Detection of hybrid individuals would require the examination of multiple nuclear loci, and would not be possible in eDNA samples containing pools of mixed genetic material from multiple individuals. In most cases, the salient point is whether DNA from an “invasive Asian carp” (meaning SC, BHC, or both) is present. Our newly developed markers meet this need and provide information as to which species the DNA most likely originated from.

The markers designed in this study represent an important advancement in our ability to detect DNA of SC and BHC in environmental DNA surveillance programs. Indeed, after extensive testing and comparison of various markers for eDNA detection of SC and BHC, markers developed in this study have been newly adopted for the official eDNA surveillance program by the U.S. Fish and Wildlife service (E. Monroe, pers. comm.). The new monitoring protocol involves initial screening using AC-TM1 and AC-TM3 markers. If at least one positive detection is found across the initial 8 replicates, then SC-TM4 and SC-TM5 and BH-TM1 and BH-TM2 will be used to confirm the presence of either species. The qPCR markers developed in this study have been chosen for this program because they provide increased throughput and sensitivity in comparison to previous approaches.

In summary, the genomic data and markers developed in this study vastly expand the tools available to detect eDNA of BHC and SC in North America and improve the accuracy, resolution, and throughput of eDNA monitoring programs. These improved tools will undoubtedly increase the accuracy and efficiency of invasive species management programs for SC and BHC in the future.

## Supporting Information

S1 Table qPCR Standard Curve Data. Slope, Y-intercept and *R* ^*2*^ values used to generate standard curves for quantifying DNA concentrations in sensitivity and multiplex qPCR reactions.
(DOCX)

S2 Table Field Testing Results (SC Markers). Patterns of positive detections in eDNA field trials (carp-infested waters from Steele Bayou) for silver carp markers designed in this study, the QAPP-SC marker, and combinations of markers to investigate how to achieve the highest rate of detections.
(DOCX)

S3 Table Field Testing Results (BH Markers). Patterns of positive detections in eDNA field trials (carp-infested waters from Steele Bayou) for bighead carp markers designed in this study, the QAPP-BH marker, and combinations of markers to investigate how to achieve the highest rate of detections.
(DOCX)

S4 Table Field Testing Results (AC Markers). Patterns of positive detections in eDNA field trials (carp-infested waters from Steele Bayou) for the AC markers designed in this study, the QAPP-SC and QAPP-BH markers, and all markers combined, and combinations of markers to investigate how to achieve the highest rate of detections.
(DOCX)
