# Supplementary material for: Mitochondrial Genome Sequencing and Development of Genetic Markers for the Detection of DNA of Invasive Bighead and Silver Carp (Hypophthalmichthys nobilis and H. molitrix) in Environmental Water Samples from the United States
Source: PLoS One. 2015 Feb 23;10(2):e0117803. doi: 10.1371/journal.pone.0117803 (PMC4338309; doi:10.1371/journal.pone.0117803)
Supplement: S1 Table — Slope, Y-intercept and R 2 values used to generate standard curves for quantifying DNA concentrations in sensitivity and multiplex qPCR reactions. (DOCX) [file pone.0117803.s001.docx]

**Table S1. qPCR Standard Curve Data.** Slope, Y-intercept and *R^2^* values used to generate standard curves for quantifying DNA concentrations in sensitivity and multiplex qPCR reactions.

1. Standard curve data for sensitivity tests shown in Table 5

|  | Y-Intercept | | *R^2^* | Slope |
| --- | --- | --- | --- | --- |
| BH-TM1 | | 37.73 | 1.00 | -3.33 |
| BH-TM2 | | 37.90 | 1.00 | -3.31 |
| BH-TM4 | | 38.43 | 1.00 | -3.36 |
| SC-TM4 | | 40.01 | 1.00 | -3.38 |
| SC-TM5 | | 38.89 | 1.00 | -3.33 |
| AC-TM1 | | 39.41 | 0.99 | -3.39 |
| AC-TM2 | | 39.20 | 1.00 | -3.37 |
| AC-TM3 | | 37.36 | 1.00 | -3.22 |

1. Standard curve data for multiplex qPCR trials shown in Table 6. Values are shown for both individual marker reactions and multiplexed (M) reactions.

|  | Y-Intercept | *R^2^* | Slope |
| --- | --- | --- | --- |
| ACTM-1 | 39.606 | 0.999 | -3.411 |
| ACTM-3 | 37.319 | 0.999 | -3.348 |
| BHTM-1 | 38.445 | 0.998 | -3.305 |
| BHTM-2 | 38.450 | 0.999 | -3.293 |
| SCTM-4 | 38.759 | 0.999 | -3.311 |
| SCTM-5 | 40.637 | 0.998 | -3.361 |
| AC-TM1 (M) | 39.864 | 0.999 | -3.418 |
| AC-TM3 (M) | 37.438 | 0.999 | -3.326 |
| BH-TM1 (M) | 38.020 | 0.999 | -3.315 |
| BH-TM2 (M) | 38.587 | 0.998 | -3.302 |
| SC-TM4 (M) | 38.751 | 0.998 | -3.251 |
| SC-TM5 (M) | 40.839 | 0.998 | -3.319 |
