# Supplementary material for: Mitochondrial Genome Sequencing and Development of Genetic Markers for the Detection of DNA of Invasive Bighead and Silver Carp (Hypophthalmichthys nobilis and H. molitrix) in Environmental Water Samples from the United States
Source: PLoS One. 2015 Feb 23;10(2):e0117803. doi: 10.1371/journal.pone.0117803 (PMC4338309; doi:10.1371/journal.pone.0117803)
Supplement: S4 Table — Patterns of positive detections in eDNA field trials (carp-infested waters from Steele Bayou) for the AC markers designed in this study, the QAPP-SC and QAPP-BH markers, and all markers combined, and combinations of markers to investigate how to achieve the highest rate of detections. (DOCX) [file pone.0117803.s004.docx]

**Table S4. Field Testing Results (AC Markers)** Patterns of positive detections in eDNA field trials (carp-infested waters from Steele Bayou) for the AC markers designed in this study, the QAPP-SC and QAPP-BH markers, and all markers combined, and combinations of markers to investigate how to achieve the highest rate of detections.

|  | QAPP-BH | QAPP-SC | AC-6 | AC-TM1 | AC-TM2 | AC-TM3 | Samples positive for at least one AC marker | Combination of the top 2 AC markers (AC-6+AC-TM2) |
| --- | --- | --- | --- | --- | --- | --- | --- | --- |
| STB 1 |  | + |  |  | + | + | + | + |
| STB 2 |  |  |  |  |  |  |  |  |
| STB 3 |  | + | + | + | + |  | + | + |
| STB 4 |  | + | + |  | + | + | + | + |
| STB 5 |  |  |  |  |  |  |  |  |
| STB 6 |  | + |  | + |  |  | + |  |
| STB 7 |  | + | + |  | + | + | + | + |
| STB 8 |  |  | + |  | + | + | + | + |
| STB 9 |  | + | + |  | + | + | + | + |
| STB 1 |  | + | + | + |  | + | + | + |
| STB 11 |  | + | + | + | + | + | + | + |
| STB 12 |  | + | + |  | + | + | + | + |
| STB 13 |  |  |  |  | + | + | + | + |
| STB 14 |  |  | + |  |  |  | + | + |
| STB 15 |  |  |  |  |  |  |  |  |
| STB 16 |  |  |  |  |  |  |  |  |
| STB 17 |  |  |  |  |  | + | + |  |
| STB 18 |  | + | + |  | + |  | + | + |
| STB 19 |  | + | + | + | + | + | + | + |
| STB 2 |  | + | + | + | + | + | + | + |
| STB 21 |  | + |  | + | + | + | + | + |
| STB 22 |  | + | + | + | + | + | + | + |
| STB 23 |  | + | + | + | + |  | + | + |
| STB 24 |  |  | + | + | + | + | + | + |
| STB 25 |  | + | + | + |  | + | + | + |
| STB 26 |  |  |  |  |  |  | + |  |
| STB 27 |  | + | + | + | + | + | + | + |
| STB 28 |  | + | + | + | + |  | + | + |
| STB 29 |  | + | + | + | + | + | + | + |
| STB 3 |  |  | + | + |  |  | + | + |
| STB 31 |  | + | + |  |  | + | + | + |
| STB 32 |  |  |  | + | + |  | + | + |
| STB 33 |  | + | + | + | + |  | + | + |
| STB 34 |  |  |  | + | + | + | + | + |
| STB 35 |  | + | + |  |  |  | + | + |
| STB 36 |  |  | + | + | + | + | + | + |
| STB 37 |  |  |  |  | + | + | + | + |
| STB 38 |  | + | + |  |  |  | + | + |
| STB 39 |  |  | + | + | + | + | + | + |
| STB 4 |  | + | + | + | + | + | + | + |
| STB 41 |  | + | + | + | + | + | + | + |
| STB 42 |  | + | + | + | + | + | + | + |
| STB 43 |  | + | + | + | + | + | + | + |
| STB 44 |  | + |  | + | + | + | + | + |
| Total positive samples | 0 | 28 | 30 | 25 | 30 | 28 | 40 | 37 |
